# Astrocytic ceramide as possible indicator of neuroinflammation

**DOI:** 10.1186/s12974-019-1436-1

**Published:** 2019-02-25

**Authors:** Nienke M. de Wit, Sandra den Hoedt, Pilar Martinez-Martinez, Annemieke J. Rozemuller, Monique T. Mulder, Helga E. de Vries

**Affiliations:** 1 Department of Molecular Cell Biology and Immunology, Amsterdam Neuroscience, Amsterdam UMC, Vrije Universiteit Amsterdam, VU University Medical Center, PO Box 7057, 1007 MB Amsterdam, the Netherlands; 2 Department of Internal Medicine, Erasmus University Medical Center, Rotterdam, the Netherlands; 3 Department of Neuroscience, School of Mental Health and Neuroscience, Maastricht University, Maastricht, the Netherlands; 4 Department of Pathology, Amsterdam UMC, Vrije Universiteit Amsterdam, Amsterdam, the Netherlands

**Keywords:** Neuroinflammation, Neurodegenerative diseases, Sphingolipids, Ceramide, Ceramide synthase, Acid sphingomyelinase

## Abstract

**Background:**

Neurodegenerative diseases such as Alzheimer’s disease (AD), Parkinson’s disease dementia (PDD), and frontotemporal lobar dementia (FTLD) are characterized by progressive neuronal loss but differ in their underlying pathological mechanisms. However, neuroinflammation is commonly observed within these different forms of dementia. Recently, it has been suggested that an altered sphingolipid metabolism may contribute to the pathogenesis of a variety of neurodegenerative conditions. Especially ceramide, the precursor of all complex sphingolipids, is thought to be associated with pro-apoptotic cellular processes, thereby propagating neurodegeneration and neuroinflammation, although it remains unclear to what extent. The current pathological study therefore investigates whether increased levels of ceramide are associated with the degree of neuroinflammation in various neurodegenerative disorders.

**Methods:**

Immunohistochemistry was performed on human post-mortem tissue of PDD and FTLD Pick’s disease cases, which are well-characterized cases of dementia subtypes differing in their neuroinflammatory status, to assess the expression and localization of ceramide, acid sphingomyelinase, and ceramide synthase 2 and 5. In addition, we determined the concentration of sphingosine, sphingosine-1-phosphate (S1P), and ceramide species differing in their chain-length in brain homogenates of the post-mortem tissue using HPLC-MS/MS.

**Results:**

Our immunohistochemical analysis reveals that neuroinflammation is associated with increased ceramide levels in astrocytes in FTLD Pick’s disease. Moreover, the observed increase in ceramide in astrocytes correlates with the expression of ceramide synthase 5. In addition, HPLC-MS/MS analysis shows a shift in ceramide species under neuroinflammatory conditions, favoring pro-apoptotic ceramide.

**Conclusions:**

Together, these findings suggest that detected increased levels of pro-apoptotic ceramide might be a common denominator of neuroinflammation in different neurodegenerative diseases.

**Electronic supplementary material:**

The online version of this article (10.1186/s12974-019-1436-1) contains supplementary material, which is available to authorized users.

## Background

Neurodegenerative diseases are characterized by a progressive loss of neuronal integrity and function, followed by neuronal death. The consequential loss of neuronal cells negatively affects numerous functions controlled by the central nervous system (CNS), such as mobility, coordination, memory, and learning, depending on the location of the area affected [1, 2]. The major neurodegenerative disorders, like Alzheimer’s disease (AD), Parkinson’s disease (PD), and frontotemporal lobar dementia (FTLD), differ in the type of neurons that are affected and the nature of the accumulated proteins that propagate neurodegeneration. Therefore, the term neurodegenerative disease comprises a wide range of conditions varying in underlying cause and localization of the neuronal loss [3]. However, a common denominator of such devastating disorders is neuroinflammation, which is increasingly recognized as a key player in the pathogenesis of several neurodegenerative diseases [4].

Neuroinflammation describes the reactive morphology and altered function of the glial compartment and involves predominantly astrocytes and microglia [5]. During disease or injury, activated glial cells serve as both source and target of proinflammatory mediators, such as cytokines, chemokines, and reactive oxygen species. Numerous neurodegenerative diseases are associated with activated glial cells, but the inflammatory reaction is not distinguishable between the different diseases, despite the varying underlying causes [6–8]. Therefore, although the observed inflammatory glial response is presumed to be secondary to neuronal death or dysfunction, it is suggested that the activation of microglia and astrocytes contributes to the progression of the different neurodegenerative diseases. Hence, understanding the underlying mechanisms involved in neuroinflammatory processes may reveal new molecular targets for future therapy [9–11].

Accumulating evidence suggests that a deregulated sphingolipid metabolism is associated with a number of neurodegenerative diseases [12–15]. In general, sphingolipids are highly enriched in the brain and are essential for the development and maintenance of the functional integrity of the nervous system [16]. The sphingolipid metabolism consists of a complex network of highly regulated pathways producing bioactive lipids that include ceramide, sphingosine, and sphingosine 1-phosphate (S1P). In most cell types, ceramide and S1P exert adverse effects on cell survival, where primarily ceramide is implicated in promoting cellular stress and cell death [17, 18]. For instance, several studies have shown that increased levels of ceramide are implicated in the induction of neural cell death, oxidative stress, and proinflammatory gene expression [19, 20]. Therefore, maintaining a strict sphingolipid balance is of key importance for cellular survival.

Ceramide synthesis occurs via three different pathways; the salvage pathway, the sphingomyelinase pathway, and the de novo pathway [21, 22]. In the salvage pathway, complex sphingolipids are catabolized into sphingosine which can then be reused to produce ceramide [23]. In addition, ceramide can also be generated from the hydrolysis of sphingomyelin through the action of sphingomyelinases, which exist in two subtypes, namely neutral sphingomyelinase (nSMase) and acid sphingomyelinase (ASM) [24, 25]. Finally, in the human brain, five different ceramide synthase (CerS1, 2, 4, 5, and 6) enzymes synthesize ceramide in the de novo pathway. These enzymes preferentially use a relatively restricted subset of fatty acyl CoAs resulting in the synthesis of ceramides with different acyl chain lengths [26–28]. Both de novo synthesis of ceramide as well as the recycling of sphingosine into ceramide in the salvage pathway are controlled by CerS enzymes. Interestingly, the SM hydrolysis and the de novo synthesis pathway have been implicated in the production of pro-apoptotic ceramides [29–31].

We previously reported evidence indicating a role for a deregulated sphingolipid balance in the pathogenesis of AD with capillary cerebral amyloid angiopathy [32]. Specifically, we revealed that activated glial cells showed increased levels of sphingolipids that are associated with the neuroinflammatory process. Moreover, a shift in the production of the different ceramide species was observed, favoring long-chain ceramides that are involved in apoptosis. Therefore, based on our recent findings, we hypothesize that a deregulated sphingolipid metabolism, specifically related to an increase in pro-apoptotic ceramides, contributes to the observed neuroinflammatory process in a wide range of neurodegenerative diseases. To test this hypothesis, we set out to investigate the expression levels of the enzymes involved in ceramide synthesis and levels of ceramide itself in different neurodegenerative diseases, which differ in their inflammatory status. For this, we selected the inferior frontal gyrus of well-characterized patients with frontotemporal dementia Pick’s disease (FTD-Pi) and patients with Parkinson’s disease with dementia (PDD) since these are frequent forms of dementia.

## Materials and methods

### Post-mortem human brain tissue

Post-mortem human brain tissue was obtained from the Netherlands Brain Bank (NBB), Netherlands Institute for Neuroscience, Amsterdam. For this study, we selected the inferior frontal gyrus of five PDD patients, five FTD-Pi patients, and five age-matched non-demented controls. All material has been collected from donors after written informed consent for brain autopsy and use of brain tissue and clinical information for research purposes. Age, gender, post-mortem delay (PMD), Braak lb, and cause of death of all cases used in this study are listed in Table 1.

**Table 1:** Summary of patient details

Patient #	Age	Gender	PMD (h)	Braak (lb)	Cause of death
Control 1	79	M	06:30	1	CVA
Control 2	85	F	05:00	0	Multi organ failure
Control 3	85	F	04:40	1	Dehydration
Control 4	80	M	08:10	1	Unknown
Control 5	78	F	07:10	0	Euthanasia
PDD 1	80	F	04:15	4	CVA, dehydration
PDD 2	82	F	03:55	6	Dehydration, pneumonia
PDD 3	83	M	05:15	6	Pneumonia
PDD 4	81	M	05:50	5	Cachexia
PDD 5	84	M	06:30	4	Myocardial infarction
FTD-Pi 1	76	F	06:40	1	Cachexia
FTD-Pi 2	77	F	05:20	2	Complications 2nd to CVA
FTD-Pi 3	70	M	05:15	1	Aspiration pneumonia
FTD-Pi 4	70	M	06:40	1	Unknown
FTD-Pi 5	68	M	15:05	1	Aspiration pneumonia

*PDD* Parkinson’s disease dementia, *FTD*-*Pi* frontotemporal dementia Pick’s disease, *PMD* post-mortem delay, *F* female, *M* male

### Immunohistochemistry

Immunohistochemistry was performed as described previously [32]. In brief, 5 μm cryosections mounted on coated glass slides (Menzel Gläser Superfrost PLUS, Thermo Scientific, Braunschweig Germany) were air-dried and fixed in acetone for 10 min. Fixating the tissue with PFA or methanol resulted in a higher background and a more patchy staining of astrocytes, confirming the use of acetone as fixative (Additional file 1). Next, the sections were incubated overnight at 4 °C with primary antibodies against ASM, ceramide, human leukocyte antigen (HLA)-DR, and glial fibrillary acidic protein (GFAP) (Table 2). All antibodies were diluted in phosphate-buffered saline (PBS) supplemented with 1% bovine serum albumin (BSA; Roche diagnostics GmbH, Mannhei, Germany). The sections were subsequently incubated with Real EnVision HRP rabbit/mouse (Dako, Glostrup, Denmark) for 30 min. Peroxidase labeling was visualized using 3,3-diaminobenzidine (DAB) as chromogen (Dako, Glostrup, Denmark). Nuclei were counterstained with hematoxylin. Finally, sections were dehydrated and mounted using Entallan (Merck, Darmstadt, Germany) after which they were analyzed with a light microscope (AXIO Scope A1, Carl Zeiss, Germany).

**Table 2:** Primary antibodies

Primary antibody	Clone	Dilution	Species raised in	Source
Ceramide	MID 15B4	1:100	Mouse	Alexis, Lausen, Switzerland [33]
ASM		1:200	Rabbit	Santa Cruz, California, USA [33]
CerS5		1:500	Rabbit	Abcam, Cambridge, UK
CerS2		1:500	Rabbit	Abcam, Cambridge, UK
GFAP		1:2000	Rabbit	DAKO, Glostrup, Denmark
LN3		1:500	Mouse	VUmc, Amsterdam, The Netherlands
GFAP-Cy3		1:300	Mouse	Sigma, MO, USA
LN3-Alexa 488		1:400	Mouse	Vumc, Amsterdam, The Netherlands

For colocalization studies, sections were incubated for 30 min containing 10% normal goat serum. Subsequently, sections were incubated overnight at 4 °C with primary antibodies as indicated in Table 2. Alexa 488-labeled goat anti-rabbit was used to detect ASM, CerS5, and CerS2 and Alexa 647-labeled goat anti-mouse was used to detect ceramide (dilution 1:400, Life Technologies). Sections were incubated for 1 h with their specific secondary antibody. Finally, sections were stained with Hoechst (dilution 1:1000, Molecular Probes) to visualize cellular nuclei and mounted with Mowiol mounting medium. The representative images were taken using a Leica TCS SP8 confocal laser-scanning microscope (Leica SP8, Mannheim, Germany), 63× oil objective. Controls of the secondary antibodies can be seen in Additional file 2.

### Quantitative and correlation analysis

Quantitative and correlation analysis of the immunohistochemical levels of ceramide, HLA-DR, GFAP, and CerS5 was performed on the gray matter of the occipital cortex of control, PDD, and FTD-Pi cases. Of each case, four pictures spanning all cortical layers of the gray matter of the inferior frontal gyrus were taken. The area fraction of the DAB staining and double-fluorescent staining was quantified using ImageJ version 1.52c.

### Lipid extraction

Sphingolipids were analyzed as previously described [32, 34, 35]. Frozen fresh human brain samples were weighed and homogenized in cold purified Millipore water (MQ, 18.2 MΩ cm) from a Milli-Q® PF Plus system (Millipore B.V., Amsterdam, the Netherlands). Total lipids were extracted from brain homogenates by adding methanol (MeOH), containing Cer-C17:0, Cer-C17:0/24:1, and S1P-D7 (2, 2, 0.2 μg/ml in methanol, respectively, Avanti Polar Lipids) as internal standard, and 10% TEA solution (trimethylamine (10/90, *v*/*v*) in MeOH/dichloromethane (DCM) (50/50, *v*/*v*)). Samples were vortexed and MeOH/DCM (50/50, *v*/*v*) was added. After 30 min at 4 °C under constant agitation, samples were centrifuged at 14,000 rpm for 20 min at 4 °C. Supernatant was transferred to a glass vial, freeze-dried, and reconstituted in MeOH before liquid chromatography-tandem mass spectrometry (LC-MSMS).

### Protein content

Protein content of all brain samples was determined by bicinchoninic acid assay (Thermo Fisher Scientific, Waltham, Massachusetts, USA) according to the manufacturer’s instructions. An 8-point calibration curve was used to determine exact protein levels. Protein content was used to normalize brain sphingolipid levels for actual input.

### LC-MSMS measurements

An autosampler (Shimadzu, Kyoto, Japan) injected 10 μL lipid extracts into a Shimadzu HPLC system (Shimadzu) equipped with a Kinetex C8 column (50 × 2.1 mm, 2.6 μm, Phenomenex, Maarssen, the Netherlands) at 30 °C using a gradient, starting from 95% mobile phase A (MQ/MeOH (50/50, *v*/*v*) containing 1.5 mM ammonium formate and 0.1% formic acid) for 2 min and increased to 93% mobile phase B (100% MeOH containing 1 mM ammonium formate and 0.1% formic acid) at 5.5 min. After 10 min, the column was flushed with 99% mobile phase B for 2 min followed by a 2-min re-equilibration. The flow rate was set at 0.25 ml/min with a total run time of 14 min. The effluent was directed to a Sciex Qtrap 5500 quadruple mass spectrometer (AB Sciex Inc., Thornhill, Ontario, Canada) and analyzed in positive ion mode following electrospray ionization.

Nine-point calibration curves were constructed by plotting area under the curve for each calibration standard Cer-C14:0, Cer-C16:0, Cer-C18:0, Cer-C20:0, Cer-C22:0, Cer-C24:1, Cer-C24:0, S1P, and SPH (Avanti polar lipids, Alabaster, AL, USA) normalized to the internal standard. Correlation coefficients (*R*^2^) obtained were > 0.999. Sphingolipid concentrations were determined by fitting the identified sphingolipid species to these standard curves based on acyl-chain length. Instrument control and quantitation of spectral data was performed using Analyst 1.4.2 and MultiQuant software (AB Sciex Inc.). Concentration of sphingolipids is expressed as pmol/mg protein.

### Real-time quantitative PCR

Human astrocytes of cortical origin, derived from fetal material ranging from 18 to 21 weeks’ gestation, were obtained from ScienCell (San Diego, CA USA) and maintained in astrocyte medium (ScienCell, San Diego, CA, USA). RNA was isolated using Trizol (Invitrogen, Carlsbad, CA, USA) according to the manufacturer’s protocol. mRNA concentrations were measured using Nanodrop (Nanodrop Technologies, Wilmington, DE, USA). cDNA was synthesized with the Reverse Transcription System kit (Promega, Madison, WI, USA) following manufacturer’s guidelines. Quantitative PCR (qPCR) reactions were performed in the Viia7 sequence detection system using the SYBR Green method (Applied Biosystems, Foster City, CA, USA). All oligonucleotides were synthesized by OcimumBiosolutions (OcimumBiosolutions, IJsselstein, The Netherlands). The primer sequence used is given in Table 3. Obtained mRNA expression levels were normalized to glyceraldehyde 3-phosphate dehydrogenase (GAPDH).

**Table 3:** Primers used for RT-qPCR

Gene	Forward primer	Reverse primer
CerS1	ACGCTACGCTATACATGGACAC	AGGAGGAGACGATGAGGATGAG
CerS2	CCGATTACCTGCTGGAGTCAG	GGCGAAGACGATGAAGATGTTG
CerS4	CTTCGTGGCGGTCATCCTG	TGTAACAGCAGCACCAGAGAG
CerS5	GCCATCGGAGGAATCAGGAC	GCCAGCACTGTCGGATGTC
CerS6	GGGATCTTAGCCTGGTTCTGG	GCCTCCTCCGTGTTCTTCAG
GAPDH	CCATGTTCGTCATGGGTGTG	GGTGCTAAGCAGTTGGTGGTG

### Statistical analysis

Statistical analysis was performed using Graphpad Prism software. Results are shown as mean with standard error of the mean. The non-parametric Kruskal-Wallis method with Dunn’s multiple comparison correction was used. Pearson correlation coefficient was calculated to evaluate the correlations between different variables.

## Results

### Neuroinflammation is associated with increased ceramide levels in astrocytes in FTD Pick’s disease

**Fig. 1:**
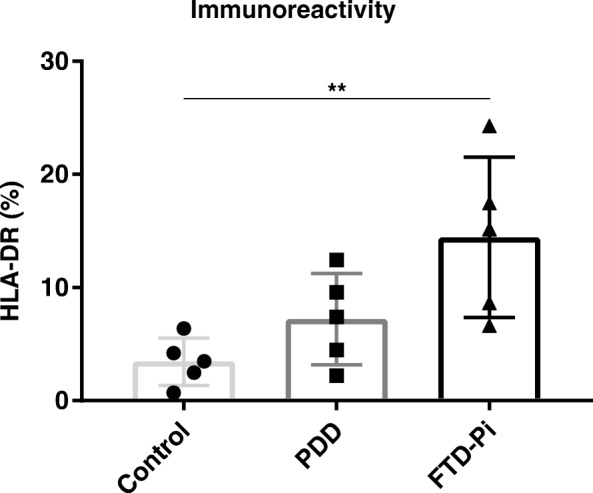
Increased activation of microglia in FTD-Pi. Quantitative analysis of the immunoreactive area for microglia (HLA-DR) in control (*n* = 5), PDD (*n* = 5), and FTD-Pi (*n* = 5). The values represent the mean ± S.E.M. Statistical significance (Kruskal-Wallis test, with Dunn’s multiple correction) indicated with asterisks: **p < 0.01

We first examined the degree of neuroinflammation in FTD-Pi, PDD, and non-demented control cases to confirm the expected difference in neuroinflammation between the three groups. Figure 1 shows a trend in increasing activation of microglia between the different groups, where a significant difference (*p* = 0.0094) is found between FTD-Pi and non-demented controls.

**Fig. 2:**
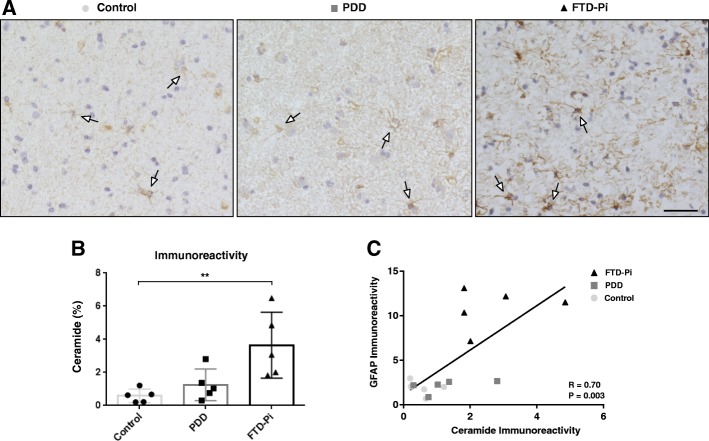
Increased levels of ceramide in FTD-Pi. **a** Immunohistochemical staining for ceramide in the inferior frontal gyrus of non-demented controls, PDD, and FTD-Pi. DAB (brown) was used as chromogen and hematoxylin (blue) was used for counterstaining of the nucleus. White arrows indicate astrocytes. Bar: 50 μm. **b** Quantitative analysis of the immunoreactive area for ceramide in FTD-Pi (*n* = 5), PDD (*n* = 5), and controls (*n* = 5). The values represent the mean ± S.E.M. **c** Correlation analysis of the immunoreactivity levels of ceramide with astrocytes (GFAP). Statistical significance (Kruskal-Wallis test, with Dunn’s multiple correction) indicated with asterisks: **p < 0.01

We next investigated the level of ceramide using immunohistochemistry. Immunohistochemical analysis of the brain sections in the gray matter revealed that specifically astrocytes showed increased immunoreactivity for ceramide (Fig. 2a). Quantification of ceramide immunoreactivity indicated a significant difference between the three groups (control: 0.58%, PDD: 1.25%, FTD-Pi: 3.65%, *p* = 0.0029). Post-hoc analysis showed a significant increase exclusively in the percentage of immunopositive areas for ceramide in FTD-Pi pathological cases compared with non-demented controls (*p* = 0.0047) (Fig. 2b).

**Fig. 3:**
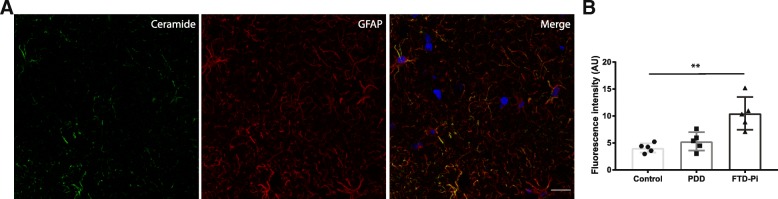
Increased levels of ceramide in specifically astrocytes in FTD-Pi. Colocalization studies indicated that **a** ceramide containing cells (green) were immunopositive for GFAP (red), indicative of astrocytes. Nuclei were counterstained with Hoechst (blue). Bar: 20 μm. **b** Quantitative analysis of the fluorescent intensity of ceramide in astrocytes in control (*n* = 5), PDD (*n* = 5), and FTD-Pi (*n* = 5). The values represent the mean ± S.E.M. Statistical significance (Kruskal-Wallis test, with Dunn’s multiple correction) indicated with asterisks: **p < 0.01

**Fig. 4:**
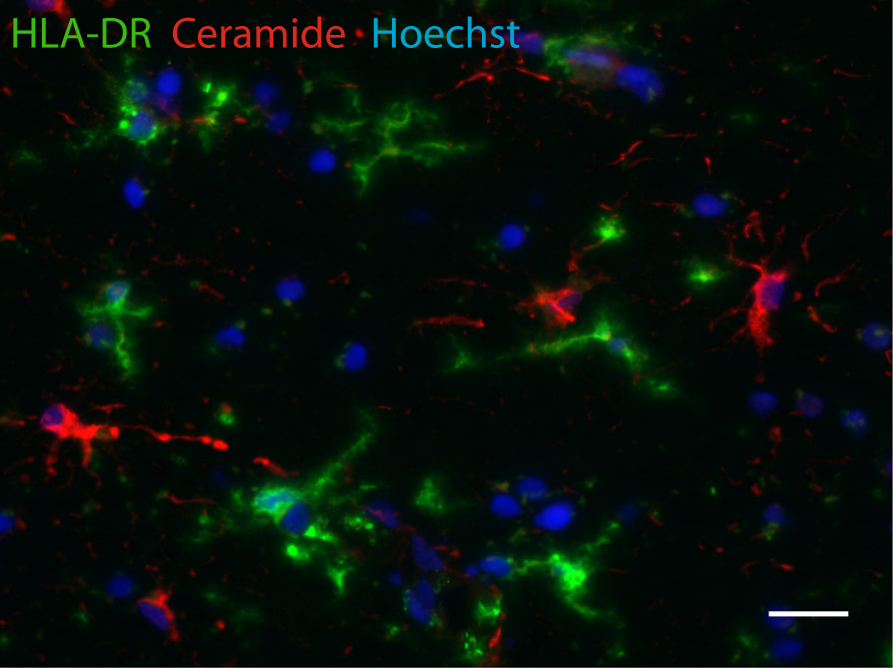
Ceramide does not colocalize with microglia. Colocalization studies indicated that ceramide containing cells (red) were not immunopositive for HLA-DR (green), indicative for microglia. Nuclei were counterstained with Hoechst (blue). Bar: 20 μm

To support the correlation between increased immunoreactivity for ceramide in astrocytes, we compared the immunoreactive levels of ceramide with an astrocytic marker (GFAP). This analysis showed a significant correlation between the expression of ceramide and GFAP (Pearson’s *r* = 0.7, *p* = 0.003) (Fig. 2c). Moreover, double immunofluorescent labeling confirmed that ceramide is specific for astrocytes (Fig. 3a) and not microglia (Fig. 4). In addition, the level of ceramide in astrocytes is significantly increased, independent of the number of astrocytes, in FTD-Pi compared to non-demented controls (*p* = 0.0037) (Fig. 3b).

### The level of ceramide in astrocytes is not regulated by acid sphingomyelinase

**Fig. 5:**
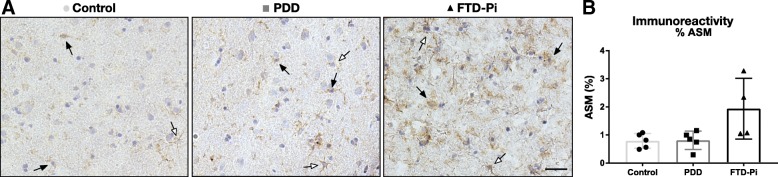
ASM expression is not specific for astrocytes. **a** Immunohistochemical staining for ASM in the inferior frontal gyrus of non-demented controls, PDD, and FTD-Pi. DAB (brown) was used as chromogen and hematoxylin (blue) was used for counterstaining of the nucleus. Black arrows indicate microglia. White arrows indicate astrocytes. Bar: 50 μm. **b** Quantitative analysis of the immunoreactive area for ASM in FTD-Pi (*n* = 4), PDD (*n* = 5), and controls (*n* = 5). The values represent the mean ± S.E.M

To gain more insight into the pathway that is responsible for the increase in ceramide observed in astrocytes, we examined the expression of the ceramide synthesizing enzyme acid sphingomyelinase (ASM) in the cortex of PDD, FTD-Pi, and non-demented age-matched controls. Immunohistochemical analysis revealed a heterogeneous image where mainly microglia showed immunoreactivity for ASM but also neurons and astrocytes, albeit to a lesser extent (Fig. 5a). The overall quantification of ASM expression did not show a significant difference between the three groups (control: 0.79%, PDD: 0.81%, FTD-Pi: 1.93%, *p* = 0.072) (Fig. 5b).

### The expression of ceramide synthase 5 in astrocytes correlates with the increase in ceramide

**Fig. 6:**
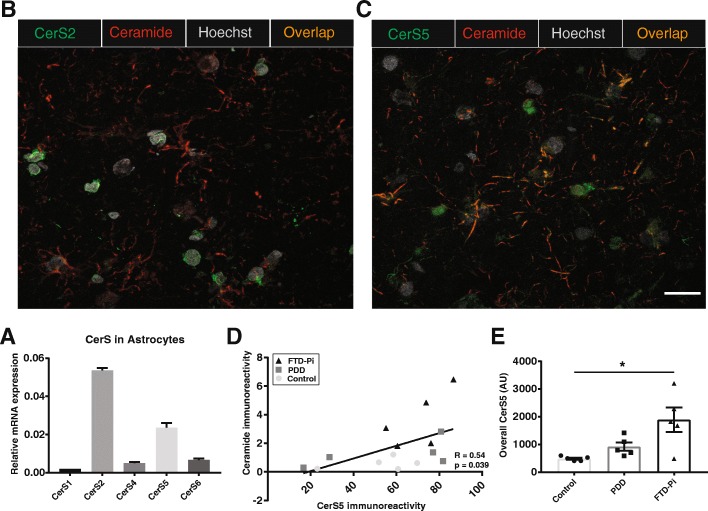
The expression of ceramide synthase 5, and not 2, in astrocytes correlates with the increase of ceramide in astrocytes. **a** The relative abundance normalized to GAPDH of the different CerSs in astrocytes where CerS2 and CerS5 relative mRNA expression are the highest. **b** Immunofluorescent double labeling of CerS2 (green) and ceramide (red) shows no colocalization, whereas **c** CerS5 (green) does colocalize with ceramide (red). Nuclei were counterstained with Hoechst (gray). Bar: 20 μm. **d** Correlation analysis of the immunoreactivity levels of ceramide with CerS5 expressed in astrocytes. **e** Quantitative analysis of CerS5 in control (*n* = 5), PDD (*n* = 5), and FTD-Pi (*n* = 5). The values represent the mean ± S.E.M, *p < 0.05

Next, we examined whether an altered regulation of ceramide synthases might be responsible for the observed enhanced ceramide levels in astrocytes. To investigate which CerS are expressed by astrocytes, we performed qPCR on isolated primary human astrocytes. Figure 6a shows that human astrocytes mainly express mRNA transcripts encoding for CerS2 and CerS5. Subsequent immunohistochemical analysis of the gray matter of PDD, FTD-Pi, and non-demented control cases revealed that CerS2 is predominantly expressed around the nuclei of different cell types, including astrocytes. However, CerS2 expression did not colocalize with GFAP or with ceramide (Fig. 6b). In contrast, immunohistochemical analysis of the gray matter of PDD, FTD-Pi, and non-demented control cases showed that predominantly reactive astrocytes express CerS5. Moreover, the CerS5 expression in astrocytes colocalized with ceramide (Fig. 6c). In addition, by quantifying the expression level of CerS5 in astrocytes using double immunofluorescent labeling, we were able to find a positive correlation between the immunoreactive ceramide levels and CerS5 expressed in reactive astrocytes (*R* = 0.54, *p* = 0.039) (Fig. 6d). Interestingly, the overall expression of CerS5 in the gray matter, independent of cell type, showed a significant increase in FTD-Pi compared to non-demented controls (*p* = 0.015)(Fig. 6e).

### C16:0 ceramide is increased under neuroinflammatory conditions in FTD Pick’s disease

**Fig. 7:**
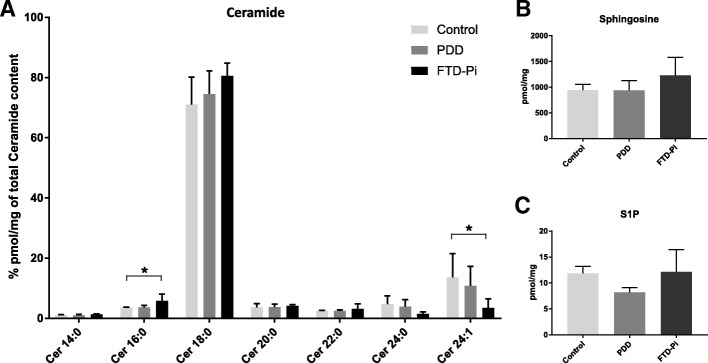
Altered ceramide levels in FTD-Pi compared to controls. **a** Levels of ceramides of different chain-lengths, **b** sphingosine, and **c** S1P were quantified by use of HPLC MS/MS in brain homogenates of FTD-Pi (*n* = 5), PDD (*n* = 5), and controls (*n* = 5). Data show the mean percentage ± S.E.M. Statistical significance between groups (Kruskal-Wallis test, with Dunn’s multiple correction) is indicated with asterisk: *p < 0.05

Finally, we investigated the ceramide content in post-mortem tissues of patients with PDD and FTD-Pi compared to non-demented controls using HPLC MS/MS to gain more insight in the alterations of the different ceramide species. Total ceramide content was not significantly different between the three groups (controls = 17,891 ± 1776, PDD = 16,376 ± 1626, and FTD-Pi = 19,644 ± 2748, *p* = 0.566). In order to compare the distribution of ceramide fatty acyl chains in the control, PDD, and FTD-Pi samples, data were expressed as a percentage of total ceramide content (Fig. 7a). Importantly, this analysis revealed a significant increase in C16:0 acyl chain content (*p* = 0.0116) accompanied by a significant decrease in C24:1 acyl chain content (*p* = 0.0392) in FTD-Pi compared to non-demented controls. To obtain a complete overview of the sphingolipid rheostat, we measured sphingosine and S1P as well (Fig. 7b, c). However, no significant differences were found for the detected levels of sphingosine and S1P between FTD-Pi, PDD, and non-demented controls.

## Discussion

In this pathological study, we set out to investigate whether increased pro-apoptotic ceramide is a common denominator of neuroinflammation in two different types of neurodegenerative diseases. The present study indicates that predominantly astrocytes show increased levels of ceramide under neuroinflammatory conditions in FTD Pick’s disease. When investigating the machinery responsible for ceramide production, no differences in the expression of ASM nor CerS2 was found, while on the other hand a positive correlation between the expression of CerS5 and ceramide in astrocytes was observed. In line with these results, a significant increase in C16:0 ceramide was observed in post-mortem tissues of FTD-Pi cases compared to non-demented controls. Previous studies from our lab and others already indicated enhanced production of ceramide by reactive astrocytes in multiple sclerosis, Alzheimer’s disease (AD), and AD with capillary cerebral amyloid angiopathy, diseases which are all characterized by various levels of neuroinflammation [32, 33, 36]. Taken together, these results are suggesting that ceramide in reactive astrocytes is a possible indicator of neuroinflammation, despite the varying underlying causes of the diseases.

We further investigated the expression of relevant enzymes in sphingolipid biology that are implicated as mediators in the cell stress response and intimately linked to inflammation [37, 38]. Unexpectedly, our data suggests that the observed ceramide production in reactive astrocytes is independent of enhanced levels of ASM. Indeed, both ASM and nSMase can act on sphingomyelin to generate ceramide. For instance, it has been shown that nSMase activity in astrocytes is quickly upregulated after cerebral ischemia [39]. However, astrocytes isolated from multiple sclerosis lesions showed increased mRNA expression of ASM linked to increased ceramide levels [33]. Therefore, the activation of the different sphingomyelinases seems to depend on disease-specific stimuli.

Interestingly, our results indicate for the first time that the increase in the expression of CerS5, the enzyme mainly responsible for the generation of C16:0 ceramide, correlates with ceramide production in astrocytes. In contrast, no correlation nor colocalization of CerS2 (main producer of C24:0 ceramide) with ceramide in reactive astrocytes was found, suggesting the ceramide in reactive astrocytes to be C16:0 ceramide and not C24:0 ceramide. Although CerS5 has been extensively studied due to its ability to synthesize C16:0 ceramide, these studies are not performed in the brain and are not addressing its role in neurodegenerative disorders [38, 40, 41]. So far, only a few studies describe the expression of CerS genes in the brain. However, most of them are either focused on different CerSs, performed in mouse brain, or are limited to analyses of their mRNA levels [42–45]. Importantly, since CerS5 is closely related to CerS6 and shows a very high extent of amino acid sequence identity, we cannot exclude the involvement of CerS6. These two enzymes share their substrate specificity for C16:0 acyl-CoA and are similar in their expression pattern [46]. Therefore, future studies are needed to identify the importance of CerS5 as ceramide-producing enzyme in astrocytes.

The correlation of CerS5 with ceramide in astrocytes is further supported by the detected increase in levels of C16:0 ceramide in FTD-Pi using HPLC MS/MS analysis of the brain homogenates. Considering that our data show that sphingosine and S1P levels do not differ between FTD-Pi and PDD compared to non-demented controls, our findings suggests that the increase of C16:0 ceramide is a result of ceramide accumulation instead of decreased ceramide breakdown. Moreover, the levels of C16:0 ceramide are inversely correlated to levels of C24:1 ceramide, indicating a shift to a more pro-apoptotic environment. It has become apparent that the different lengths of the fatty acids of ceramide exert a variety of distinct functions, where for instance C16:0 ceramide has been suggested to be the dominant species elevated during apoptosis [47–49]. Several studies related to cancer report opposing effects of the long-chain ceramides (C14:0–C20:0) compared to the very-long-chain ceramides (C22:0–C26:0), where a shift from very-long to long-chain ceramides increases apoptosis, suggesting the equilibrium between the chain lengths of ceramides is regulating cell death [50, 51]. However, the direct association found in these studies cannot be concluded from our results since these studies were performed in cell lines, whereas our HPLC MS/MS measurements include whole brain tissue homogenates, comprising different cell types. Therefore, we can only conclude that the sphingolipid balance is altered but cannot yet pinpoint which cell type(s) are responsible for this change.

The increased ceramide levels in astrocytes might serve as a possible therapeutic target to limit ongoing neuroinflammation. Not only ceramide but also the ceramide-derived glycosphingolipid lactosylceramide in astrocytes is increased during chronic CNS inflammation and promotes inflammation and neurodegeneration [52]. Moreover, ceramide can be secreted by astrocytes and subsequently affect neighboring cells, possibly acting as a mediator for neuronal apoptosis [53, 54]. Upon activation, astrocytes show an upregulation of the sphingosine-1-phospate receptor 3 (S1P3) in different neurodegenerative diseases [32, 55]. It has been shown that S1P3 is a target of Fingolimod, a synthetic analog of S1P, which is approved as an oral treatment for relapsing-remitting multiple sclerosis [56]. Previous studies indicate that Fingolimod administration reduces ceramide formation in reactive astrocytes [33]. In addition, the secreted ceramide might also act as a possible biomarker for neuroinflammation in neurodegenerative disorders. For instance, in the cerebral spinal fluid of patients with MS, an increase in certain ceramide species was found [57]. Also, studies are ongoing to identify individuals at increased risk of cognitive impairment by characterizing distinct plasma ceramides profiles. Interestingly, promising preliminary results showed increased C16:0 levels in plasma of PDD patients, indicating a possible predictive value for ceramide [13].

Importantly, there are some limitations to our study, such as the relatively low number of unique well-characterized cases, which renders caution in the interpretation of the data. However, through the combination of a variety of validated methods, we were still able to demonstrate significant differences between the different groups. Inclusion of more cases may reduce the chance of a type 2 error, but having found an effect, albeit the limited statistical power, makes us confident on the relevance of our data. In addition, although PDD as an intermediate group did show a trend in all our results, increasing the number might push it to significance. Also, the isolation of astrocytes was not possible with our tissue, hence we can only speculate about the increase of C16:0 ceramide as the product of CerS5 in astrocytes. Therefore, the identification of CerS5 as possible initiator of the production of C16:0 ceramide in astrocytes would benefit from an additional mechanistic study.

## Conclusion

In conclusion, the present results suggest that astrocytic ceramide is closely associated with the neuroinflammatory process. Importantly, we identified CerS5 as possible mediator of C16:0 ceramide in astrocytes in the human brain. While only a relatively small sample size was feasible in this study, the significant differences that were found in the sphingolipid balance between the groups provide useful avenues for further research and possible future treatments, based on modifying the sphingolipid pathway.

## Additional files

Additional file 1: Comparison of different fixation methods that show less optimal staining for ceramide. (PDF 312 kb)

Additional file 2: Control staining using only the secondary antibodies envision, goat anti-rabbit Alexa 488 (green), and goat anti-mouse Alexa 647 (red). (PDF 151 kb)

## Abbreviations

ASM: Acid sphingomyelinase
CerS: Ceramide synthase
FTD-Pi: Frontotemporal dementia Pick’s disease
GFAP: Glial fibrillary acidic protein
PDD: Parkinson’s disease with dementia
S1P: Sphingosine-1-phosphate
