# Supplementary figures and images for: Astrocytic ceramide as possible indicator of neuroinflammation
Source: J Neuroinflammation. 2019 Feb 25;16:48. doi: 10.1186/s12974-019-1436-1 (PMC6388480; doi:10.1186/s12974-019-1436-1)

**Methanol fixation**

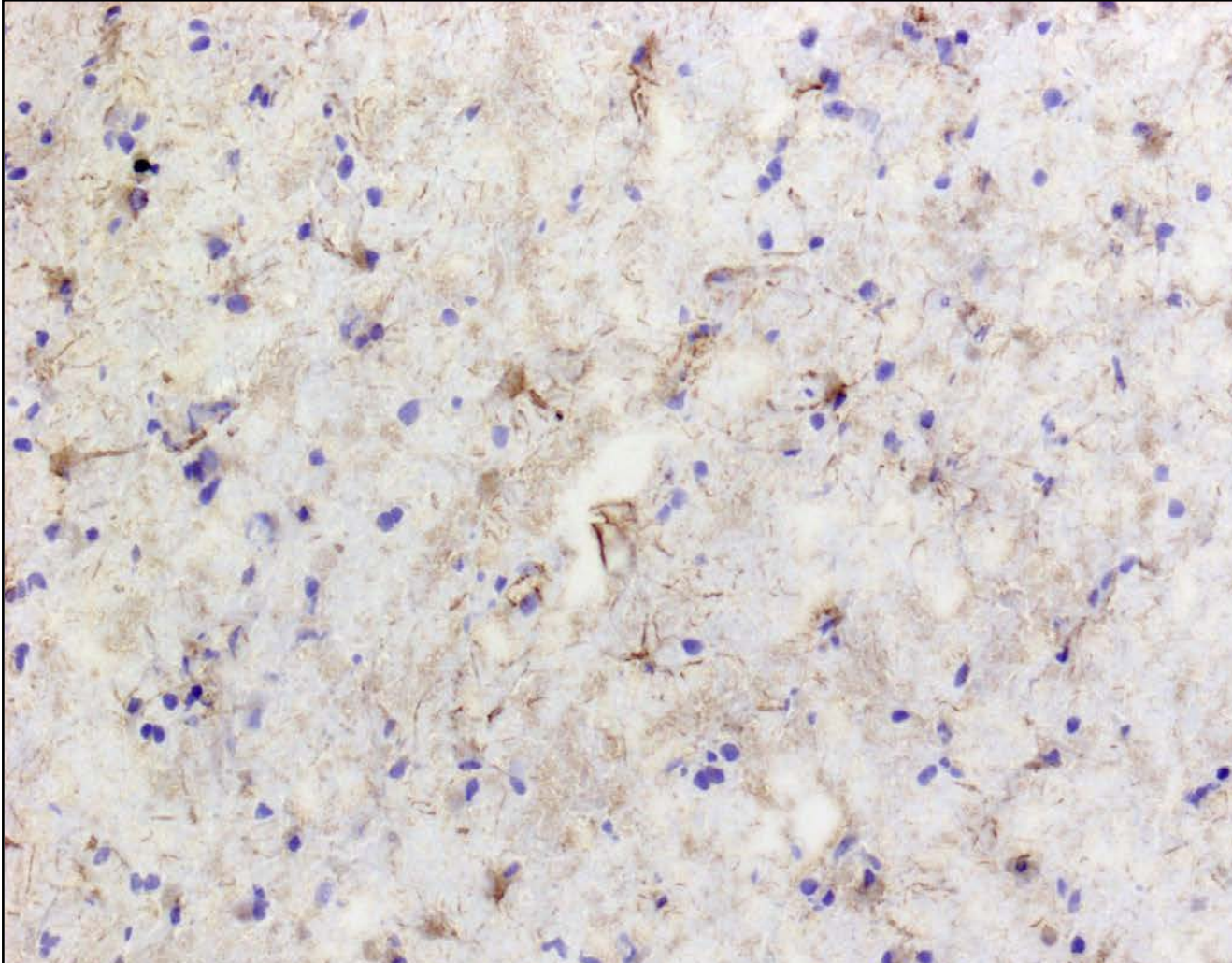

**4%PFA fixation**

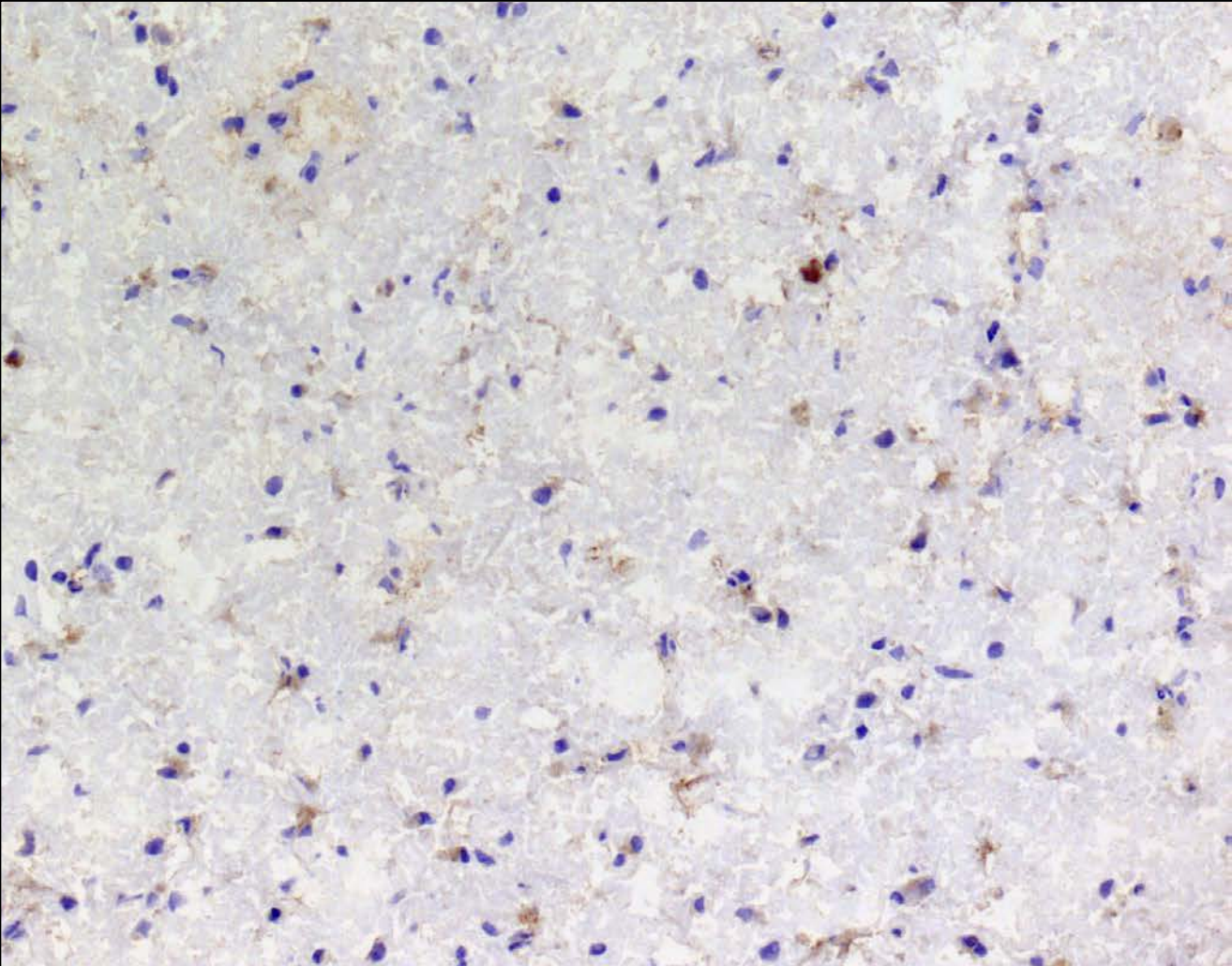

Supplement: Supplementary file 1 — Comparison of different fixation methods that show less optimal staining for ceramide. (PDF 312 kb) [file 12974_2019_1436_MOESM1_ESM.pdf]

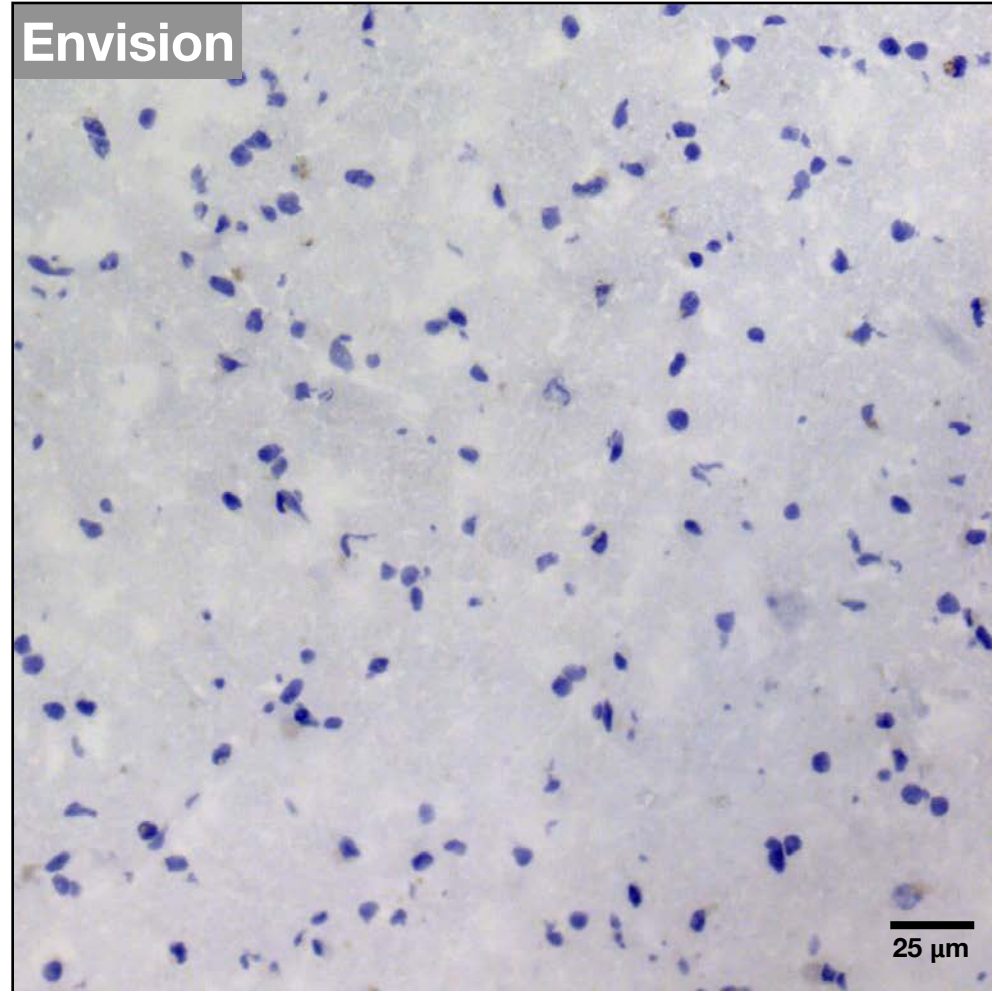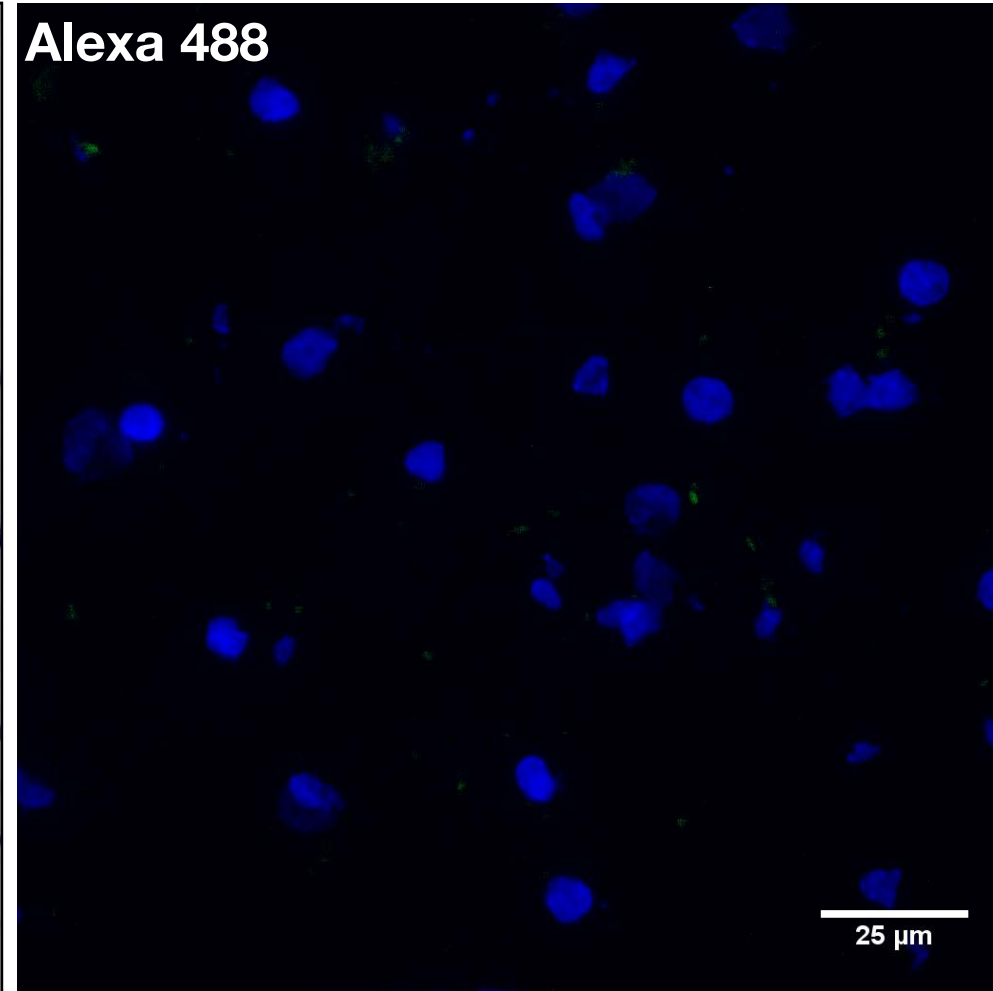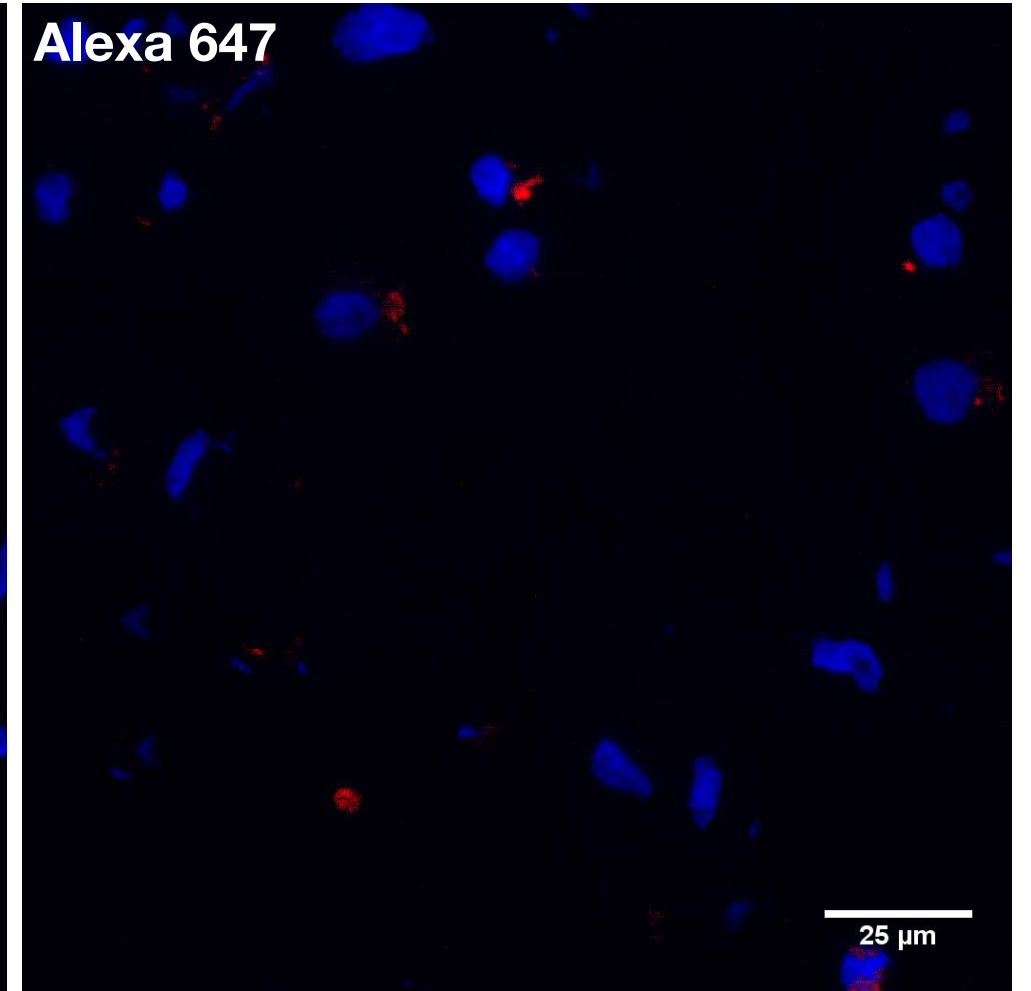

Supplement: Supplementary file 2 — Control staining using only the secondary antibodies envision, goat anti-rabbit Alexa 488 (green), and goat anti-mouse Alexa 647 (red). (PDF 151 kb) [file 12974_2019_1436_MOESM2_ESM.pdf]
